# The validity of the Youth Physical Activity Questionnaire in 12–13-year-old Scottish adolescents

**DOI:** 10.1136/bmjsem-2016-000163

**Published:** 2017-01-06

**Authors:** Paul Robert Walker McCrorie, Ana Perez, Anne Ellaway

**Affiliations:** MRC/CSO Social and Public Health Sciences Unit, University of Glasgow, Glasgow, Scotland

**Keywords:** Physical activity, adolescents, validity, self-report, accelerometry

## Abstract

**Background:**

The development of accurate methods to measure health-behaviours forms an integral component in behavioural epidemiology. Population surveillance of physical activity often relies on self/proxy reported questionnaires due to cost and relative ease of administration. The aim of this study was to examine the criterion validity and measurement agreement between the Youth Physical Activity Questionnaire (YPAQ) and accelerometry before being included in a Scotland-wide study.

**Methods:**

Forty four participants (12–13 years old; 61% girls) completed the YPAQ following 7 days wearing the Actigraph GT3X+. Mean moderate-to-vigorous physical activity (MVPA) per day was derived from YPAQ and accelerometer and validity was assessed using Spearman's correlation; Bland-Altman plots examined absolute agreement between methods.

**Results:**

Pearson's and Spearman’s correlations between YPAQ and accelerometer were r = 0.47 and r_s_ = 0.39 (p<0.01) respectively. The YPAQ over reported mean MVPA by 25.6 ± 50.2 minutes (95% CI 10.4-40.9 minutes; p <0.001), with 95% limits of agreement of −72.69 minutes and + 123.99 minutes. Evidence of underreporting at lower levels of activity and over reporting at higher levels of activity was evident (Pearson's r=0.81), in addition to heteroscedasticity, where variances increased as MVPA increased.

**Conclusions:**

Although a moderate correlation between the two methods was apparent, the YPAQ should not be used interchangeably with accelerometry. The YPAQ does demonstrate a reasonable ability to rank MVPA, although it tends to under-report lower levels and over-report higher levels. This, and other administering factors, should be taken into consideration if being used for group or individual level analyses.

**What are the new findings?**

- The YPAQ demonstrates reasonable criterion validity metrics in 12–13 year old Scottish adolescents. Although confirming previous validity coefficients in English adolescents, this is the first YPAQ validity study of Scottish adolescents.
- Bland Altman plots demonstrate poor agreement and a tendency for the YPAQ to under- report moderate to vigorous physical activity at lower levels of activity and over-report at higher levels of activity.
- A greater level of measurement error is introduced as activity levels increase.
- YPAQ should not be used interchangeably with accelerometry, and the employment of this questionnaire in a population setting needs careful consideration.

## Background

The development of accurate methods to measure health behaviours forms an integral component in behavioural epidemiology.^1^ Within physical activity (PA) research, high quality measures are crucial in all stages of the research process, including population surveillance. Accelerometry and movement sensors have become a widely used objective method for quantifying PA levels through their ability to derive information relating to frequency, duration and intensity of PA from actual body movement/acceleration. Although successfully integrated into large-scale studies,^2^ only a few population level datasets exist using this particular method. Self- or proxy-reported questionnaires remain popular, despite a number of limitations:^3 4^ questionnaire responses depend on perception, encoding, storage and retrieval of information;^5^ and concerns exist over the accuracy of questionnaire data from children under 10 years due to their cognitive underdevelopment.^6^ These concerns translate to poor validity coefficients,^7^ where a tendency exists for questionnaires to over-report PA levels compared with directly measured PA.^8^ However, within population surveillance research, a questionnaire approach requires less technical knowledge and expertise and is considered less burdensome than accelerometry. Although cheaper and more practical to administer,^9^ questionnaires can be adapted more readily to different delivery methods (ie, postal or face-to face administration), have been developed to suit different population groups (eg, children, adolescents),^10^ and, finally, they have the ability to extract information on ‘type’ (eg, sporting activities, play or active living) and ‘domain’ (eg, school, home, commute) of activity. For these reasons, it is important that they accurately measure the activity being studied. The Youth Physical Activity Questionnaire (YPAQ)^11^ is based on the Children’s Leisure Activities Study Survey (CLASS);^12^ and measures frequency, duration, intensity and mode, over the past 7 days, of both PA and sedentary activities throughout all domains. Original validation work was conducted in England with 12–13-year-olds and demonstrated acceptable levels of validity compared with accelerometry (r_s_=0.42, p=0.04). With reasonable measurement properties, and with the ability to capture the multiple components of PA, it was decided to employ the YPAQ for this study, specifically within a Scottish population. Prior to commencing a large-scale, country-wide data collection (Studying Physical Activity in Children's Environments across Scotland (SPACES)), employing both objective and self-reported measures of PA to estimate the prevalence of children meeting the UK Chief Medical Officer's PA guidelines,^13^ we set out to examine the ability of the YPAQ to accurately capture the main outcome variable used to assess guideline adherence, namely moderate-to-vigorous physical activity (MVPA). Specifically, we examined the individual level criterion validity and measurement agreement between YPAQ-derived MVPA and accelerometry-derived MVPA (ActiGraph GT3X+; ActiGraph LLC, Pensacola, FL, USA) to assess the suitability of YPAQ to measure this outcome variable for inclusion in the SPACES study.

## Methods

### Participants

A convenience sample of 90 adolescents (12–13 years old) from two schools in Central/West Scotland were invited to take part. Participants were automatically enrolled (following participant assent) in the study unless parents withdrew consent (opt out consent).

Ethical approval for the study was granted by the University of Glasgow’s College of Social Sciences, the participating school’s local educational authorities and the head teachers of both schools. The study fieldwork was conducted in May 2013 and included three school days, two weekend days and 2 days which fell on public holidays.

### Measures

#### Objective measurement: accelerometer

PA was measured using an accelerometer (ActiGraph GT3X+) worn on a belt around the waist for seven consecutive days. The GT3X+ is a small (4.6×3.3×1.5 cm), lightweight (19 g), tri-axial device that records and stores raw acceleration signals in three axes, at a user-specified sample rate (between 30 and 100 Hz). It has a dynamic range of ±6 G and memory capacity of 512MB. ActiGraph devices are used extensively, and internationally, in children’s PA research;^2 14 15^ the GT3X+ has been validated against indirect calorimetry in children’s energy expenditure research.^16^

Following data collection, ActiGraph data were uploaded to a computer for post-processing using ActiGraph’s proprietary software (ActiLife, v6.7.1). PA files were trimmed to include only the measurement period. The software aggregated the raw acceleration data (100 Hz) into 30-second epochs. Periods of 60 consecutive zeros, allowing for ‘spikes’ of 2 min of activity (less than 100 counts/min), were classified as non-wear and subsequently removed in any PA outcome measure. Participants had to wear the device for 500 min for it to be classified as a valid day,^9^ and a minimum of three valid days were required for inclusion in the analyses.^17 18^ MVPA per valid day per participant was extracted using the Evenson threshold (counts per minute >2295) cut points.^19^ Mean MVPA was calculated per participant (as a function of number of valid days per participant), and then across the full sample.

#### Self-reported questionnaire: YPAQ

The YPAQ contains 47 different activities and requests participants to report the frequency and duration of each activity for both weekdays and weekend days over the past 7 days. The YPAQ is broken into contextual settings/domains: sporting, leisure, school and free-time activities.^11^

On completion of the accelerometer protocol (on day 8), participants attended a large classroom, where trained fieldworkers assisted with the completion of the YPAQ over an allocated school period (55 min). The fieldworkers read the instructions, showed an example of how a question should be filled out and allowed the pupils to ask questions before starting. Upon completion, fieldworkers were instructed to check for errors or omissions (eg, missed questions, illegible/ambiguous answers).

#### Scoring

Each activity in the questionnaire was assigned a metabolic equivalent (MET) value according to previously published values.^20^ For the purposes of this study, activities with values above 4 METs were considered to be at least moderate and included in the analysis.^21^ The activities included cricket, dancing, football, gymnastics, martial arts and rugby. Mean time per day in MVPA was calculated per participant (derived from the total MET minutes divided by seven) and then across the group.

#### Statistical analyses

The null hypothesis that no bias exists between measurement methods (YPAQ vs accelerometer) was initially tested using a paired t-test. The strength of the association between both measures was tested using Pearson's correlation and Spearman's rank correlation. A Bland-Altman plot,^22^ showing mean bias and 95% limits of agreement was used to assess the degree of absolute agreement between methods, and differences between measurements were calculated for each participant (YPAQ−accelerometer) and plotted against the mean of each method ((YPAQ+accelerometer)/2). The relationship between these differences (YPAQ−accelerometer) and the mean was tested using a Pearson correlation. This provided an indication of the dependency of the differences on the underlying measurement range.

Considering accelerometry to be the criterion method, the values representing the differences (YPAQ−accelerometer) were plotted against the accelerometer (figure 3A). Potential heteroscedasticity across the range of MVPA (accelerometer) was assessed by conducting a Breusch-Pagan/Cook-Weisberg test:^23^ visually represented by plotting the residuals versus predicted values (figure 3B).

All statistical analyses were performed using Stata version 13 (Stata Corp, College Station, TX, USA).

## Results

Of the original 90 participants invited, 7 opted out prior to the study commencing. A further six withdrew their consent during the study, four were absent during data collection and two accelerometers were lost during the monitoring period, leaving 71 participants who took part in the full data collection period. Forty-four participants (61% girls) provided at least three valid days of PA and were included in the agreement analyses. The mean age was 12.7 years.

### Mean PA levels

On average, children spent 58.2±20.3 min per day in MVPA according to accelerometry, with boys spending approximately 1.3 more minutes per day in MVPA than girls. Self-reported time spent in MVPA was much higher than that recorded by accelerometer, with an average time of 99.8±56.2 min per day, with boys reporting on average 29.3 more minutes in MVPA than girls (table 1).

**Table 1:** Baseline characteristics and time spent in MVPA (both methods)

MVPA (min)	Girls (n=27)	Boys (n=17)	Overall (n=44)
Age (years)	12.7	12.8	12.7
Accelerometer	57.68±22.07	58.96±17.71	58.18±20.28
YPAQ	88.44±55.08	117.76±54.83	99.77±56.23

MVPA, moderate-to-vigorous physical activity; YPAQ, Youth Physical Activity Questionnaire.

### Validity coefficients

Pearson's and Spearman’s correlations between YPAQ and accelerometer were r=0.47 and r_s_=0.39 (p<0.01), respectively, indicating a statistically significant medium monotonic relationship between the two methods.

The mean difference in minutes spent in MVPA between YPAQ and accelerometer was 25.6±50.2 min (95% CI 10.4 to 40.9; p<0.001); the mean difference between methods was more pronounced among boys (table 2).

**Table 2:** Measurement differences in time spent in MVPA: YPAQ−accelerometer

	Average difference (min)	95% CI	p Value*
Girls	13.75±45.32	−4.2 to 31.7	0.20
Boys	44.5±53.0	17.3 to 71.8	<0.001
Overall	25.6±50.2	10.4 to 40.9	<0.001

*Wilcoxon signed-ranked test.

MVPA, moderate-to-vigorous physical activity; YPAQ, Youth Physical Activity Questionnaire.

### Agreement between methods

**Figure 1:**
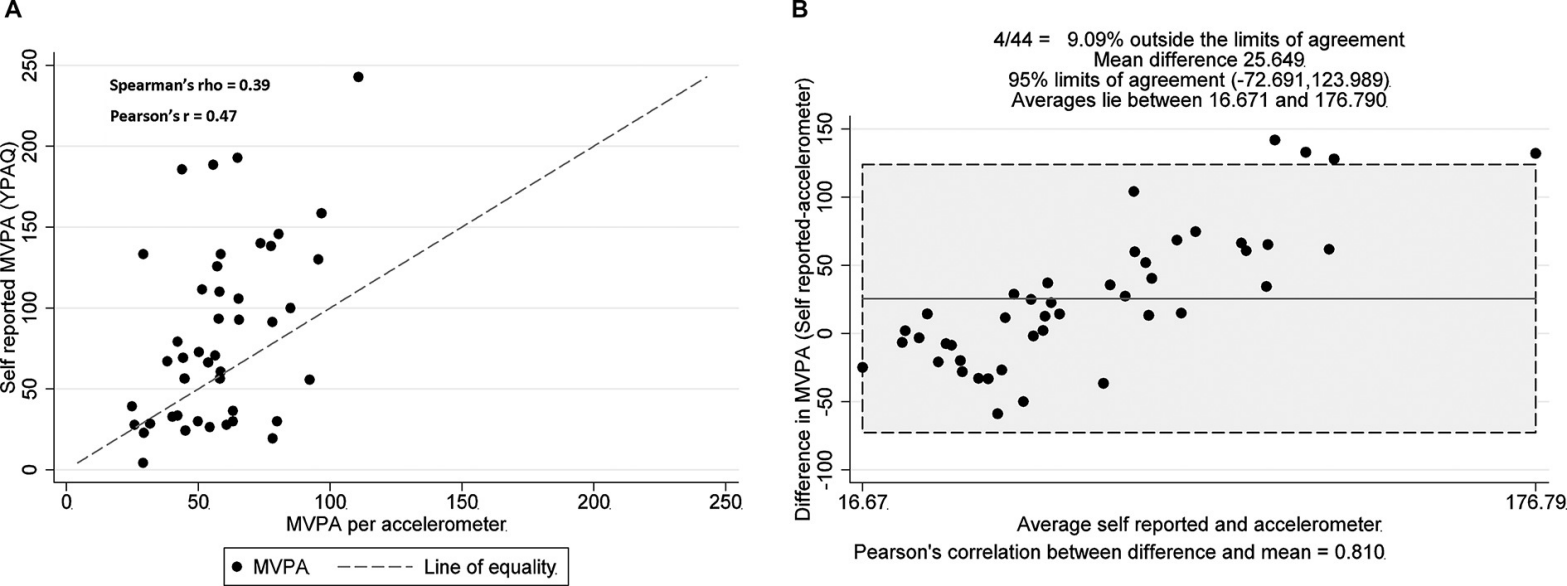
(A) Individual participant data points plotting YPAQ-derived MVPA versus accelerometer-derived MVPA. (B) Bland-Altman plot with mean bias and 95% limits of agreement (noted by shaded section and upper and lower dashed lines). MVPA, moderate-to-vigorous physical activity; YPAQ, Youth Physical Activity Questionnaire.

Figure 1A demonstrates that data points do not fall on the line of equality (perfect agreement) across levels of measurement. This initial plot illustrates that YPAQ scores tend to be greater than accelerometer-derived MVPA, with a slight trend in the bias: being negative (YPAQ scores lower) for lower levels of accelerometer-derived MVPA and positive for high levels of accelerometer-derived MVPA.

**Figure 2:**
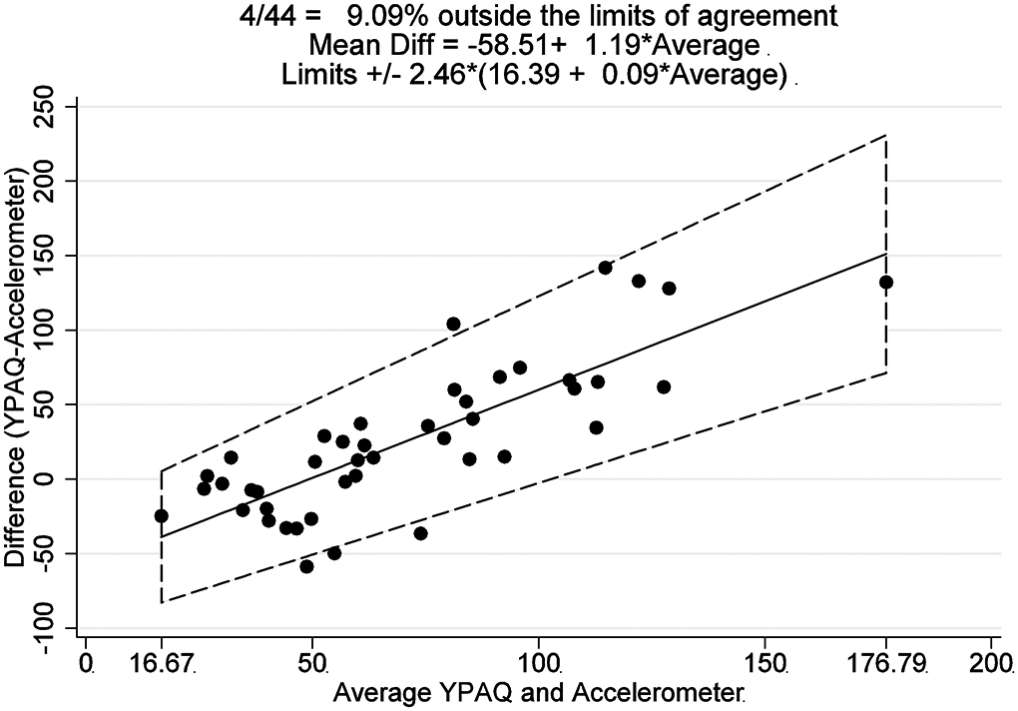
Mean bias and 95% limits of agreement using a regression approach for non-uniform differences.

The Bland-Altman plot (figure 1B) identified a mean bias between the methods of 25.65 min of MVPA, with 95% limits of agreement of −72.69 and +123.99 min (YPAQ−accelerometer). There is evidence of both under- and over-reporting, dependent on the mean level of MVPA. The differences tended to be negative when mean MVPA was low and positive when mean MVPA was high. Pearson's correlation between the difference and the mean was 0.81, indicating a significant positive linear relationship between these two variables. Where there are instances of a relationship of this magnitude, Bland and Altman^24^ suggest a regression approach for non-uniform differences (figure 2). Using this approach, the limits are slightly narrower at lower levels of MVPA and widen as MVPA increases.

**Figure 3:**
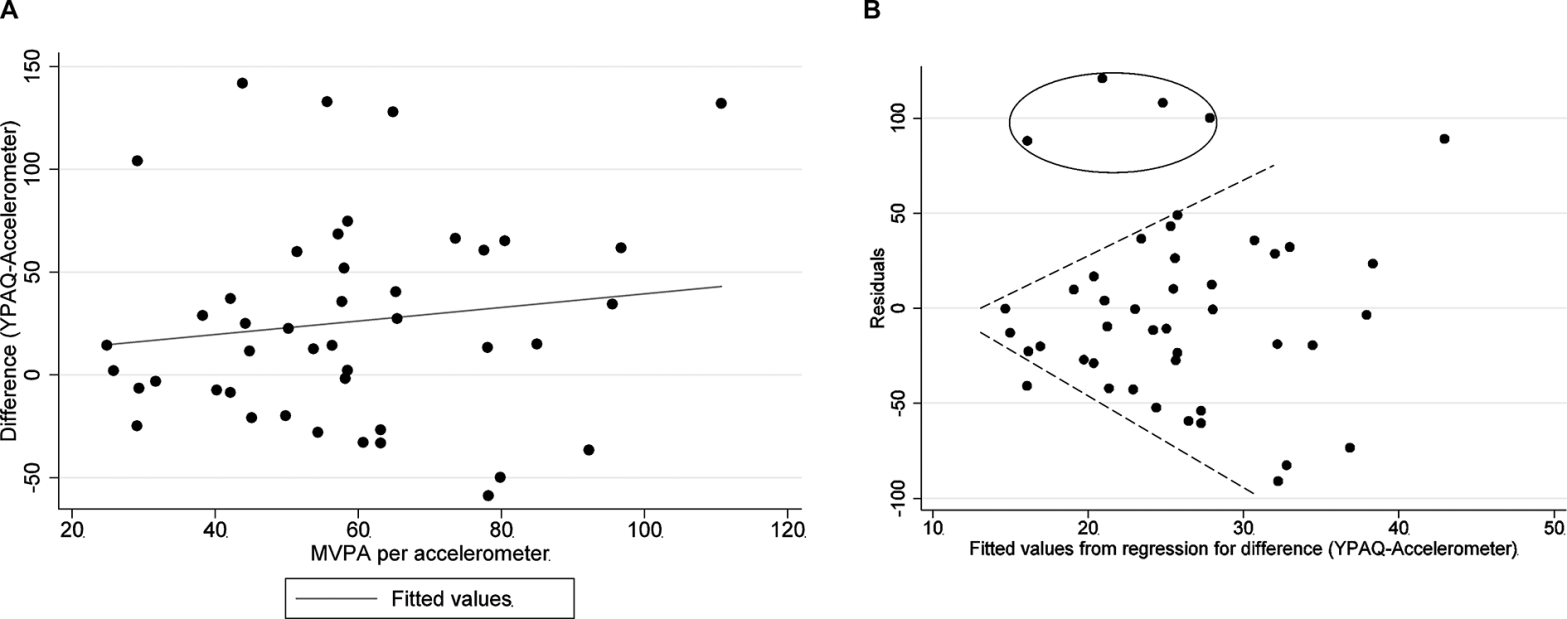
(A) Scatter plot representing the difference between YPAQ-derived MVPA and accelerometer-derived MVPA plotted against the criterion method (accelerometer); the regression line applied indicates that residuals increase as MVPA increases. (B) Fitted values from figure 3A versus residuals; this plot visually confirms a tendency for greater measurement error at higher levels of MVPA. MVPA, moderate-to-vigorous physical activity; YPAQ, Youth Physical Activity Questionnaire.

The Breusch-Pagan/Cook-Weisberg test (figure 3A and B) was conducted to test for constant variance of residuals across predicted values (of differences between measurement methods). This led us to accept the null hypothesis that all error variances were equal (p=0.2899). However, once influential cases and outliers (figure 3B, circled data points) were identified and removed, there was evidence of heteroscedasticity, as shown in figure 3B (the variance increases as the values increase).

## Conclusions

### Interpretation of findings

The main purpose of the analysis was to investigate the validity metrics of the YPAQ as a self-reported measure for extracting time spent in MVPA in young adolescents as compared with accelerometry. In the event of its acceptability, the measure could be translated to testing in the population setting where its purpose would be to estimate the population prevalence of children meeting the PA guidelines. The results demonstrated that a moderate linear correlation existed between methods (Pearson's r=0.48; Spearman’s r_s_=0.39), although results from the Bland-Altman analysis demonstrated a poor level of agreement, with error between measures dependent on the underlying PA level (r=0.81).

We were interested in determining whether the YPAQ would be a valid proxy for accelerometry, given that questionnaires could be considered more practical for population surveillance than activity monitors. When used to assess the prevalence of children meeting PA guidelines, Sallis and Saelens^25^ have stated the importance of measuring absolute levels of validity. As can be seen from our findings, the agreement between the two methods becomes less evident as MVPA increases (error and overestimation increases), effectively widening the limits of agreement. The YPAQ, although demonstrating acceptable validity through correlational metrics, including the ability to rank individuals' PA, shows systematic bias through the measurement range as demonstrated by the Bland-Altman analyses. We would therefore advise caution if it is used to extract accurate levels of MVPA to be used in population prevalence estimates.

### Comparisons with the original validation work

The initial validation work undertaken by Corder and colleagues^11^ was conducted using a population group (12–13 year olds; n=25) similar to that of the present study (12–13 year olds; n=44). Compared with participants in our study, those in the Corder study recorded 14 min/day more in MVPA (72 vs 58 min) as measured by accelerometry; median MVPA by YPAQ in the Corder sample was 92 min/day compared with 100 min/day in our sample. The differences in accelerometry can be explained, to some degree at least, by the particular cut point used in each method (>1952 vs >2295 counts/min), although the use of different ActiGraph models, epoch length from which the MVPA was calculated and processing options, such as non-wear time and total valid time per day, will have also contributed to these differences. YPAQ scores were similar in both studies, indicating some consistency in the measure conducted across different samples and in different years (2005–2006 and 2013–2014). Furthermore, we found a similar relationship between questionnaire and accelerometer when ranking the data (Spearman’s r_s_ of 0.42 in the Corder study vs 0.39 in the current study). This finding provides some support for the ability of the YPAQ to rank PA levels at an individual level.

Both studies demonstrated a general over-reporting of MVPA by the YPAQ although a stronger, and significant, bias was found in the present study; 22.4 min MVPA/week (95% CI −155.6 to 200.4) in the Corder sample compared with 25.6 min/day (95% CI 10.4 to 40.9) in the present study. Only our study found that the degree of questionnaire error was dependent on activity level (Pearson correlation of 0.81 vs 0.02), with the complicated pattern observed suggesting under-reporting at lower levels of activity and over-reporting at higher levels. The dependence of error across the measurement range is seldom reported in the literature,^7^ but it can be seen from the present study that this finding may be considered problematic if it is used in population surveillance studies where guideline prevalence is of key importance.

### Comparisons with other literature

The literature supports the premise that self-reported/indirect measures of PA may over-report activity levels. In a systematic review conducted by Adamo and colleagues,^8^ it was found that 72% of the reviewed indirect measures overestimated the directly measured values. Within the same review, correlations ranged from −0.56 to 0.89 highlighting both negative and positive relationships between the measures. In contrast to our findings, the review by Adamo and colleagues reported that girls were more likely to overestimate direct values of PA than boys(by 584% vs 114%, girls and boys, respectively). Why we have observed the opposite pattern is unclear, although one potential explanation may be that football and running were more commonly recorded among boys—often with large and extreme values.

The ability of the YPAQ to successfully rank MVPA is supported by a number of recent reviews on self-reported measures.^7 26^ However, having the ability to rank PA is different to its ability to be used accurately as a surrogate for PA prevalence. Helmerhorst and colleagues in their recent review suggested that ‘despite considerable effort, accurate and precise self-report physical activity instruments are still scarce’.^7^ The reduction of a complex multidimensional construct (PA) into a single metric, potentially misleading understandings of what criterion methods are and, importantly, a lack of a comprehensive measurement framework have been cited as potential reasons for the inconsistencies seen in the literature.^27^ We have to consider the participants themselves when we discuss inconsistency. Feedback from our fieldworkers suggested that many participants struggled with the concept of frequency and duration of activities. Cognitive immaturity, including memory recall, and the comprehension of questionnaire content can be problematic in youth.^25^ Future work will collect data from 10-year-old children, and will be self-administered rather than interviewer/fieldwork administered. Our experience in this study—with older children—suggests that issues may arise over comprehension of the YPAQ, and consequently affect data quality. A recent review^28^ assessed 89 PA measures for their applicability to population surveillance and identified a small group of measures that received scientific and expert support. One of these measures, the Physical Activity Questionnaire for Children (PAQ-C)^10^ may address the memory and comprehension issues faced within this study by assessing general levels of PA rather than trying to ascertain all facets of the behaviour.

### Strengths and limitations

This study endeavoured to replicate the original design conducted by Corder and colleagues,^11^ including similar participant ages and statistical approach. A strong scientific approach, particularly in measurement studies, is one where previous work is replicated, challenged, supported or refuted. This is especially true when employing different population groups in different settings. By doing so, greater confidence in the YPAQ's validation properties can be expected. As advocated in the literature,^29^ we have been clear with regard to the measurement purpose, derivation of our outcome and analysis. Furthermore, this study tried to improve data quality by allocating significant time and resources to the process, including the employment of fieldworkers to actively supervise questionnaire completion.

One limitation of our study is that our sample size lacked the power to detect subgroup differences (eg, by gender), as 52% (n=46) of the participants did not meet the accelerometry inclusion criteria. Additionally, our decision to include children with 3 days of valid PA may not have been sufficient enough to provide an accurate representation of daily PA.^30^ Moreover, these valid days may not have included a weekend day, which often involve less wear time, and PA levels, than weekdays.^31^ Therefore, compared with the YPAQ, which asks participants to recall based on the ‘previous 7 days’, we may have introduced error into the analyses and inflated the level of MVPA as measured by accelerometry. Even so, there remained a significant overestimation of the YPAQ against the accelerometer. Furthermore, the accelerometers were worn during the waking hours and removed only for water-based activities and contact activities. As such, some activities (eg, swimming or rugby) may not have been recorded. Additionally, some activities (eg, cycling) may have been misclassified due to the placement of the accelerometer. This information could be included in future studies through the addition of self-reported cycling time; doing so may reduce the size of the overestimation.

In summary, although moderately correlated, these two methods should not be used interchangeably as agreement was poor, with error in the measurement highly dependent on activity level. From a practical perspective, the face-to-face administration of the YPAQ highlighted a number of concerns, and its employment in population surveillance (where a face-to face delivery may not be possible) to extract individual level MVPA should be considered carefully. Conversely, if a suitable standardised error was identified and adjusted for, then the YPAQ could be a cheaper, more practical way to measure PA if methods were employed to improve in situ participant comprehension.

## References

[1] Sallis JF, Owen N, Fotheringham MJ Behavioral epidemiology: a systematic framework to classify phases of research on health promotion and disease prevention. Ann Behav Med 2000;22:294–8. 10.1007/BF02895665 11253440

[2] Cooper AR, Goodman A, Page AS, et al Objectively measured physical activity and sedentary time in youth: the International children's accelerometry database (ICAD). Int J Behav Nutr Phys Act 2015;12:113 10.1186/s12966-015-0274-5 26377803 PMC4574095

[3] Basterfield L, Adamson AJ, Parkinson KN, et al Surveillance of physical activity in the UK is flawed: validation of the Health Survey for England Physical Activity Questionnaire. Arch Dis Child 2008;93:1054–8. 10.1136/adc.2007.135905 18782845

[4] Dishman RK, Washburn RA, Schoeller DA Measurement of physical activity. Quest 2001;53:295–309. 10.1080/00336297.2001.10491746

[5] Durante R, Ainsworth BE The recall of physical activity: using a cognitive model of the question-answering process. Med Sci Sports Exerc 1996;28:1282–91. 10.1097/00005768-199610000-00012 8897386

[6] Sallis JF Self-report measures of children's physical activity. J Sch Health 1991;61:215–19. 10.1111/j.1746-1561.1991.tb06017.x 1943046

[7] Helmerhorst HJ, Brage S, Warren J, et al A systematic review of reliability and objective criterion-related validity of physical activity questionnaires. Int J Behav Nutr Phys Act 2012;9:103 10.1186/1479-5868-9-103 22938557 PMC3492158

[8] Adamo KB, Prince SA, Tricco AC, et al A comparison of indirect versus direct measures for assessing physical activity in the pediatric population: a systematic review. Int J Pediatr Obes 2009;4:2–27. 10.1080/17477160802315010 18720173

[9] Ridgers ND, Fairclough S Assessing free-living physical activity using accelerometry: Practical issues for researchers and practitioners. Eur J Sport Sci 2011;11:205–13. 10.1080/17461391.2010.501116

[10] Crocker PR, Bailey DA, Faulkner RA, et al Measuring general levels of physical activity: preliminary evidence for the Physical Activity Questionnaire for Older Children. Med Sci Sports Exerc 1997;29:1344–9. 10.1097/00005768-199710000-00011 9346166

[11] Corder K, van Sluijs EM, Wright A, et al Is it possible to assess free-living physical activity and energy expenditure in young people by self-report? Am J Clin Nutr 2009;89:862–70. 10.3945/ajcn.2008.26739 19144732

[12] Telford A, Salmon J, Jolley D, et al Reliability and validity of physical activity questionnaires for children: the Children’s Leisure Activities Study Survey (CLASS). Pediatr Exerc Sci 2004;16:64–78. 10.1123/pes.16.1.64 19827457

[13] Department of Health. Start Active, Stay Active: a report on physical activity from the four home countries' Chief Medical Officers. London: Crown Copyright, 2011.

[14] McCrorie PR, Fenton C, Ellaway A Combining GPS, GIS, and accelerometry to explore the physical activity and environment relationship in children and young people—a review. Int J Behav Nutr Phys Act 2014;11:93 10.1186/s12966-014-0093-0 25356782 PMC4172984

[15] Riddoch CJ, Bo Andersen L, Wedderkopp N, et al Physical activity levels and patterns of 9- and 15-yr-old European children. Med Sci Sports Exerc 2004;36:86–92. 10.1249/01.MSS.0000106174.43932.92 14707773

[16] Crouter SE, Horton M, Bassett DR Validity of ActiGraph child-specific equations during various physical activities. Med Sci Sports Exerc 2013;45:1403–9. 10.1249/MSS.0b013e318285f03b 23439413 PMC3686914

[17] Mattocks C, Ness A, Leary S, et al Use of accelerometers in a large field-based study of children: protocols, design issues, and effects on precision. J Phys Act Health 2008;5(Suppl 1):S98–111. 10.1123/jpah.5.s1.s98 18364528

[18] Møller NC, Kristensen PL, Wedderkopp N, et al Objectively measured habitual physical activity in 1997/1998 vs 2003/2004 in Danish children: the European Youth Heart Study. Scand J Med Sci Sports 2009;19:19–29. 10.1111/j.1600-0838.2008.00774.x 18282221

[19] Evenson KR, Catellier DJ, Gill K, et al Calibration of two objective measures of physical activity for children. J Sports Sci 2008;26:1557–65. 10.1080/02640410802334196 18949660

[20] Ainsworth BE, Haskell WL, Whitt MC, et al Compendium of physical activities: an update of activity codes and MET intensities. Med Sci Sports Exerc 2000;32:S498–516. 10.1097/00005768-200009001-00009 10993420

[21] Troiano RP, Berrigan D, Dodd KW, et al Physical activity in the United States measured by accelerometer. Med Sci Sports Exerc 2008;40:181–8. 10.1249/MSS.0b013e31817057da 18091006

[22] Bland JM, Altman DG Statistical methods for assessing agreement between two methods of clinical measurement. Lancet 1986;1: 307–10. 2868172

[23] Breusch TS, Pagan AR A simple test for heteroscedasticity and random coefficient variation. Econometrica 1979;47:1287–94. 10.2307/1911963

[24] Bland JM, Altman DG Measuring agreement in method comparison studies. Stat Methods Med Res 1999;8:135–60. 10.1191/096228099673819272 10501650

[25] Sallis JF, Saelens BE Assessment of physical activity by self-report: status, limitations, and future directions. Res Q Exerc Sport 2000;71:S1–14. 10.1080/02701367.2000.11082780 10925819

[26] Chinapaw MJ, Mokkink LB, van Poppel MN, et al Physical activity questionnaires for youth: a systematic review of measurement properties. Sports Med 2010;40:539–63. 10.2165/11530770-000000000-00000 20545380

[27] Kelly P, Fitzsimons C, Baker G Should we reframe how we think about physical activity and sedentary behaviour measurement? Validity and reliability reconsidered. Int J Behav Nutr Phys Act 2016;13:32 10.1186/s12966-016-0351-4 26931142 PMC4772314

[28] Biddle SJ, Gorely T, Pearson N, et al An assessment of self-reported physical activity instruments in young people for population surveillance: Project ALPHA. Int J Behav Nutr Phys Act 2011;8:1 10.1186/1479-5868-8-1 21194492 PMC3019119

[29] Ainsworth BE, Caspersen CJ, Matthews CE, et al Recommendations to improve the accuracy of estimates of physical activity derived from self report. J Phys Act Health 2012;9(Suppl 1):S76–84. 10.1123/jpah.9.s1.s76 22287451 PMC3544158

[30] Trost SG, Pate RR, Freedson PS, et al Using objective physical activity measures with youth: how many days of monitoring are needed? Med Sci Sports Exerc 2000;32:426–31. 10.1097/00005768-200002000-00025 10694127

[31] Riddoch CJ, Mattocks C, Deere K, et al Objective measurement of levels and patterns of physical activity. Arch Dis Child 2007;92: 963–9. 10.1136/adc.2006.112136 17855437 PMC2083612

